# Predicting Survival in Bevacizumab-Treated Colorectal Cancer: Personalized Mathematical Models Based on Clinical and Angiogenic Biomarkers

**DOI:** 10.3390/ijms26199332

**Published:** 2025-09-24

**Authors:** Diana Cornelia Moisuc, Mihai Vasile Marinca, Bogdan Gafton, Daniela Constantinescu, Petru Cianga, Mariana Pavel-Tanasa

**Affiliations:** 1 Department of Immunology, Grigore T. Popa University of Medicine and Pharmacy Iasi, 700115 Iasi, Romania; moisucdianac@gmail.com (D.C.M.); d.constantinescu@umfiasi.ro (D.C.); petru.cianga@umfiasi.ro (P.C.); 2 Department of Oncology, Grigore T. Popa University of Medicine and Pharmacy Iasi, 700115 Iasi, Romania; mihai.marinca@umfiasi.ro (M.V.M.); bogdan.gafton@umfiasi.ro (B.G.); 3 Department of Oncology, Regional Institute of Oncology, 700483 Iasi, Romania; 4 Immunology Laboratory, “St. Spiridon” Clinical County Hospital, 700106 Iasi, Romania

**Keywords:** bevacizumab, colorectal cancer, circulating angiogenic biomarkers, prognostic mathematical models

## Abstract

Aberrant activation of proangiogenic signaling pathways, particularly the vascular endothelial growth factor (VEGF) axis, drives neovascularization and tumor progression in colorectal cancer (CRC). Bevacizumab targets VEGF-A-mediated angiogenesis, but the lack of validated predictive biomarkers limits personalized treatment. In this prospective study, we evaluated a panel of circulating angiogenic biomarkers combined with clinical parameters, using mathematical models to predict survival in metastatic CRC patients treated with bevacizumab and chemotherapy. Low VEGF-A and VEGF-D levels, together with high bFGF, were associated with improved overall survival (OS). A logistic regression model incorporating these biomarkers, regional lymph node invasion, and primary tumor resection status showed significant prognostic accuracy (*p* < 0.001). Incorporating CypA further refined the model, identifying patients with low VEGF-A, VEGF-D, and CypA, and high VEGF-C and PlGF, as having the most favorable OS. These findings demonstrate that integrating clinical and circulating biomarker data can improve individualized risk assessment and support personalized therapeutic strategies for CRC patients receiving bevacizumab.

## 1. Introduction

Angiogenesis is a multifaceted process that plays a crucial role in the growth and progression of tumor cells. This occurs through the release of tumor-derived factors, extracellular matrix-associated cytokines, and the overexpression of pro-angiogenic mediators such as vascular endothelial growth factor (VEGF), placental growth factor (PlGF), and angiopoietins [1]. The radiological tumor response to anti-angiogenic therapies varies widely due to the heterogeneity of angiogenic mechanisms, the biological heterogeneity of tumors, and the intricate network of intracellular signaling pathways activated in response to hypoxia. Nevertheless, certain tumors eventually develop resistance to anti-angiogenic treatment [2]. In the era of personalized medicine, the identification and application of prognostic and predictive biomarkers are essential for optimizing therapeutic strategies in oncology and across various medical conditions.

A topic of growing interest in assessing the benefits of antiangiogenic therapy is the analysis of panels of circulating biomarkers in relation to progression free survival (PFS), overall survival (OS) or radiological tumor response due to the benefits of accessibility, low cost and possibility of obtaining repeated samples for serial testing. Such studies have been conducted in ovarian cancers [3], hepatocellular carcinomas [4], angiosarcomas [5], lung cancers [6], gastric cancers [7] and colorectal cancers (CRC), but the results regarding the prognostic or predictive role of circulating proangiogenic factors are contradictory [8,9,10].

In the CALGB 80405 study, a panel of 24 plasma biomarkers was measured at baseline to evaluate their prognostic and predictive significance in 715 patients with metastatic colorectal cancer (CRC) exhibiting wild-type KRAS status. Patients were randomized to receive chemotherapy in combination with either bevacizumab or cetuximab. The analyzed markers were involved in angiogenesis, as well as in inflammation and generation of the immune response [8]. Vascular endothelial growth factor A (VEGF-A) and PlGF proved to be prognostic factors for OS, but not for PFS. Elevated PlGF baseline levels were associated with a lack of PFS benefit from bevacizumab treatment, regardless of the chemotherapy regimen administered. Conversely, lower levels of VEGF-D were correlated with improved PFS outcomes, with the predictive effect being most pronounced in patients whose VEGF-D levels fell within the lowest quartile.

Interestingly, Zhang et al. investigated the expression levels of VEGF-A, along with the soluble forms of its receptors VEGFR1 (FLT-1), and VEGFR2, across two independent colon cancer datasets [9]. Their analysis revealed that elevated expression of all three markers was associated with poor prognosis. The observation was also confirmed in another dataset, where high expression levels of the three factors were specifically linked to unfavorable outcomes in patients with RAS wild-type tumors. In contrast, a study by Delle Monache et al. found no significant link between progression-free survival (PFS) and baseline levels of VEGF-A or IL-8 in patients with metastatic colorectal cancer harboring RAS mutations who were treated with bevacizumab [10].

In a recent review summarizing data from a phase II trial evaluating the efficacy of bevacizumab combined with irinotecan-based chemotherapy in metastatic CRC, the authors observed that, prior to radiological disease progression, certain subgroups of patients showed increased plasma levels of cytokines and proangiogenic factors. This increase may suggest a mechanism of resistance to antiangiogenic therapy. The authors observed an increase in PlGF values following bevacizumab administration, with the highest levels registered just before disease progression [11].

Basic Fibroblast Growth Factor (bFGF/FGF-2), is involved in promoting cell growth, angiogenesis, and modulating the tumor microenvironment [12]. However, little is known about its role in CRC. Jibiki et al. examined the clinical significance of bFGF in CRC and showed that bFGF levels differ significantly between early and advanced stage cancers, being influenced by tumor size and extent of lymphatic invasion. Patients with larger tumors and moderate lymphatic invasion show elevated bFGF levels. These data suggest a potential prognostic role of bFGF [13].

The VEGF-C/VEGFR3 signaling pathway is crucial for promoting the proliferation, migration, and survival of lymphatic endothelial cells, as well as facilitating the metastatic process [14]. In CRC, Tacconi et al. demonstrated that VEGF-C expression is upregulated, while VEGFR3 is present in both lymphatic vessels and tumor-associated macrophages. VEGF-C/VEGFR3 signaling synergically activates lymphatic endothelial cells and tumor-associated macrophages, resulting in the suppression of antitumor immunity and the enhancement of primary tumor growth [15].

Tyrosine Kinase with Immunoglobulin-like and Epidermal Growth Factor-like Domains 2 (Tie2) is the receptor of angiopoietins and is involved in the modulation of angiogenesis and vascular permeability. Abnormal activation of the Tie2 signaling pathway has been linked to excessive angiogenesis in cancer and tumor progression, thus potentially becoming a target for antiangiogenic therapy [16,17]. In metastatic CRC, the results of a study showed that after bevacizumab administration, Tie2 levels correlated with an imaging marker of the tumor vasculature, suggesting that Tie2 is secreted by the tumor vasculature. Prior to antiangiogenic therapy, Tie2 levels independently correlated with the vascular characteristics of tumors. After treatment, Tie2 levels were associated with radiological tumor response and PFS [18].

The link between cyclophilin A (CypA) and angiogenesis was investigated recent studies, which demonstrated that under hypoxic conditions, CypA is upregulated and acetylated, leading to enhanced autophagy in endothelial cells. This acetylated form of CypA was shown to promote proliferation, migration, and the formation of tubular networks in pulmonary arterial endothelial cells. These effects were accompanied by increased endothelial cell motility and angiogenic activity, highlighting a role for CypA in modulating vascular remodeling and angiogenesis in response to cellular stress [19]. Additionally, Peng et al. demonstrated that CypA plays a key role in regulating oxidative stress and the production of reactive oxygen species, mechanisms through which it contributes to the development of chemoresistance in CRC. Furthermore, increased levels of CypA were correlated with lack of radiologic response to chemotherapy, suggesting its implications in the mechanisms of treatment resistance and its potential predictive role [20]. Additionally, in a recent study, we demonstrated that CypA correlates with improved OS, being a favorable prognostic factor [21].

In our study, we propose a novel perspective on the dual prognostic and predictive roles of bevacizumab therapy initiation in CRC by simultaneously evaluating, in the same cohort of patients, a comprehensive panel of angiogenesis-related biomarkers, namely bFGF, PlGF, VEGF-A, VEGF-C, VEGF-D, FLT-1, Tie2, and CypA, alongside baseline patient characteristics, including age, primary tumor location, RAS mutational status, metastatic sites, and the associated chemotherapy regimen. While the majority of these circulating angiogenic biomarkers were assessed only at baseline, CypA measurements were available both at baseline and after six months of treatment, as previously reported in our recent study [21]. We subsequently developed mathematical prediction models integrating clinical parameters with these biomarkers to identify those with relevance for both OS and PFS. This approach enables a more personalized treatment strategy by identifying patients most likely to benefit from bevacizumab, potentially minimizing unnecessary exposure to adverse effects and reducing healthcare costs.

## 2. Results

### 2.1. Baseline Characteristics

The study included 80 patients (ITT group) who received chemotherapy and bevacizumab, treatment initiated between May 2019 and January 2021. The median age was 61 years (range 37–82) and 55% of the patients were males. The study group included 18 patients initially presenting with localized colorectal cancer, who later presented progressive disease with metastases. Upon detection of metastases, bevacizumab and chemotherapy were initiated. Most of the primary tumors (71.3%) were located on the left colon. Surgical excision of the primary tumor was performed in 70% of the patients. Metastases were most commonly localized in the liver (42.5%), although a similar proportion of patients (42.5%) had metastases at multiple sites. The most frequently used chemotherapy regimen was based on oxaliplatin (57.5%). The disease control rate was 48.8% and the objective response rate was 20%. Among the 80 patients, CypA values were available for 52, who were analyzed as a prognostic factor in our recent study. This group was labeled in the present study as the PDS—Partial Data Subset—CypA. The results regarding clinicopathological characteristics in the ITT group remain consistent in the subgroup of patients for whom CypA values are available.

The baseline characteristics of the ITT group (80 patients) and the PDS subgroup (52 patients) are presented in Table 1.

### 2.2. Treatment-Induced Adverse Effects

The most frequent adverse events identified in the ITT group are described in Table 2. Liver toxicity was the most common adverse event, followed by anemia and proteinuria, regardless of grade. Neutropenia was the most commonly reported grade 3 or higher adverse event. There were no grade 5 adverse events.

### 2.3. Clinicopathological Factors Associated with OS

**Figure 1:**
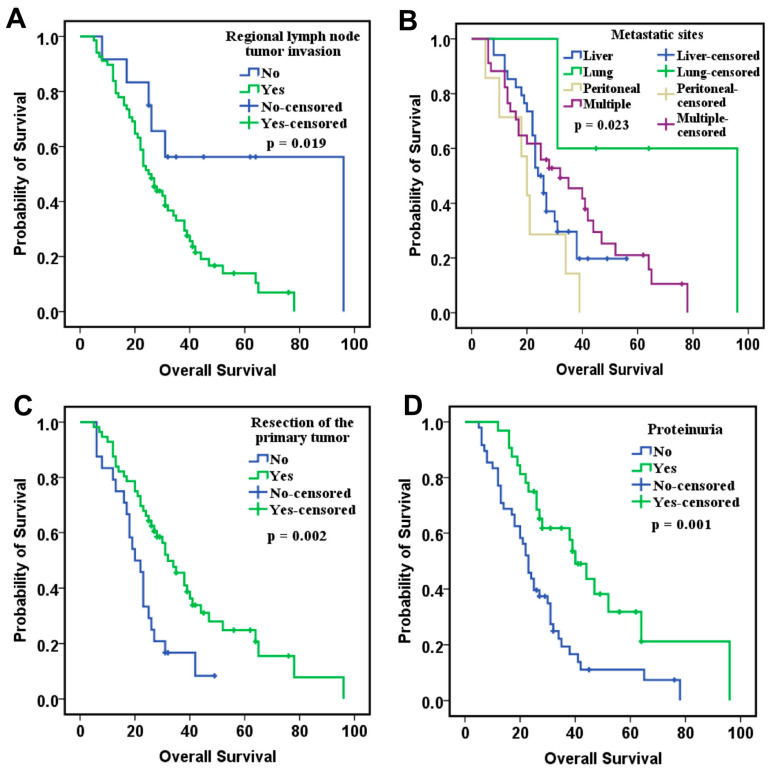
Kaplan–Meier curves of overall survival according to: (**A**) regional lymph node tumor invasion (n = 68 with invasion, n = 12 without), (**B**) metastatic sites (n = 34 liver, n = 5 lung, n = 7 peritoneal, n = 34 multiple), (**C**) primary tumor resection status (n = 56 resected, n = 24 unresected), and (**D**) treatment-induced proteinuria (n = 32 with proteinuria, n = 48 without).

To examine the association between clinicopathological factors and OS in patients with metastatic CRC, we evaluated their prognostic value using Kaplan–Meier survival curves and Log-Rank test. In the ITT group, the median OS was 27 months (range: 5–96), while the median PFS was 6 months (range: 1–37). One patient was lost to follow-up after one month of treatment. Tumor invasion of regional lymph nodes significantly influenced OS. Patients with tumor invasion of regional lymph nodes had a shorter OS (96 vs. 25 months, *p* = 0.019; Figure 1A). Additionally, the location of metastases influenced OS, with the shortest OS observed in patients with peritoneal metastases compared to those with pulmonary or multiple metastases (20 vs. 96 vs. 32 months, *p* = 0.023; Figure 1B). Patients with resected primary tumor showed an improved OS (32 vs. 20 months, *p* = 0.002; Figure 1C). Among the adverse effects identified, only the presence of proteinuria was significantly associated with OS, with patients who developed proteinuria showing better OS (40 vs. 23 months, *p* = 0.001; Figure 1D).

**Figure 2:**
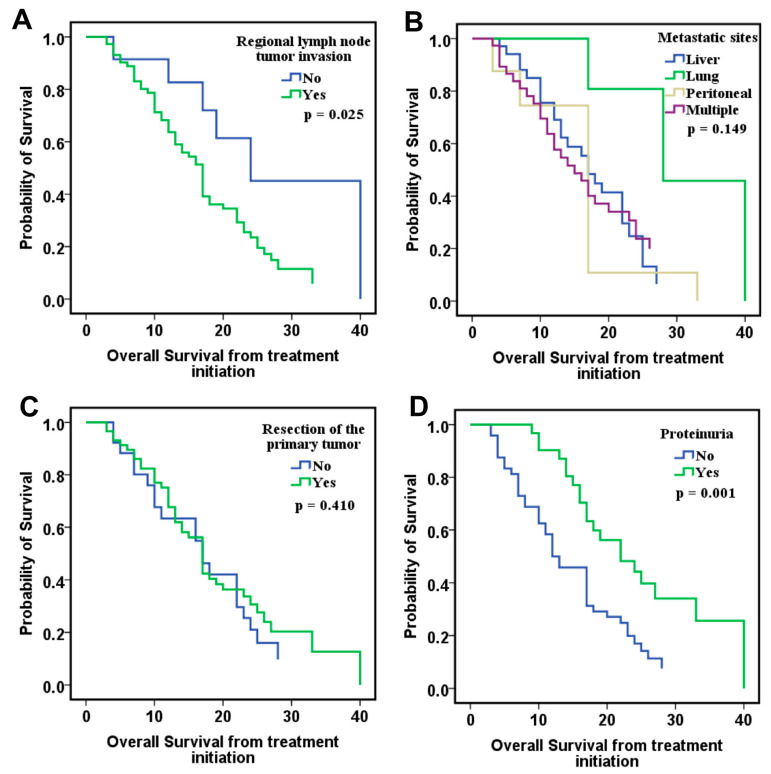
Cox regression overall survival from treatment initiation in relation to: (**A**) regional lymph node tumor invasion (n = 68 with invasion, n = 12 without), (**B**) metastatic sites (n = 34 liver, n = 5 lung, n = 7 peritoneal, n = 34 multiple), (**C**) primary tumor resection status (n = 56 resected, n = 24 unresected), and (**D**) treatment-induced proteinuria (n = 32 with proteinuria, n = 48 without).

Next, we aimed to investigate the above-mentioned parameters in relation to overall survival measured from treatment initiation (OS-TI) by Cox-regression analysis adjusted for time-dependent covariates such as proteinuria and hypertension episodes. The regional lymph node tumor invasion (*p* = 0.025) and proteinuria (*p* = 0.001) remained important factors influencing OS-TI, whereas resection of the primary tumor did not significantly affect survival (*p* = 0.410). Interestingly, patients with lung-only metastases demonstrated improved OS-TI compared to those with metastases at other sites, while no differences were observed between those with liver and peritoneal metastases (Figure 2).

### 2.4. Serum Biomarkers Analysis

#### 2.4.1. Biomarker Levels and Distribution Ranges

Table 3 provides an overview of the serum biomarker concentrations, including their median values and distribution ranges, offering insight into their variability across the study population.

An analysis of the clinicopathological factors revealed statistically significant differences in the levels of the analyzed markers, influenced by the location and excision of the primary tumor, as well as the number of involved regional lymph nodes. We observed a significant association between the location of the primary tumor and VEGF-D values. Patients with tumors in the left colon were more likely to have extremely high levels of VEGF-D, whereas those with tumors in the right colon predominantly had low levels (*p* = 0.007, Table 4). Additionally, Tie2 expression was higher in patients with tumors on the left colon. This could indicate biological differences between tumors located on the right and left sides of the colon. On the other hand, lower Tie2 levels were observed in patients with lymph node tumor involvement, suggesting a significant association between reduced Tie2 expression and regional lymph node involvement (*p* = 0.030). Moreover, we observed a trend where patients who have undergone primary tumor resection more often exhibit high Tie2 levels, while those who have not undergone surgery tend to have lower levels. However, this association does not reach statistical significance (*p* = 0.051) but may become significant in a larger study cohort.

#### 2.4.2. The Prognostic Role of Biomarkers

None of the analyzed biomarkers were statistically significant when assessed individually in relation to OS, being classified into high or low values according to the median values for each biomarker (Figure S1). Since we identified clinicopathological factors that are associated with OS, we further investigated whether biomarkers influence OS, adjusting for these factors using multivariate Cox analysis. In the analyzed model, all previously evaluated factors maintained their prognostic significance for OS, except for metastatic sites. Interestingly, the biomarkers VEGF-A, VEGF-D and bFGF correlated with OS. For patients without regional node involvement at diagnosis, those with primary tumor resection, and those who developed proteinuria during treatment, these three biomarkers become prognostic factors. Patients with low levels of VEGF-A and VEGF-D and high levels of bFGF had the best prognosis (Table 5).

**Figure 3:**
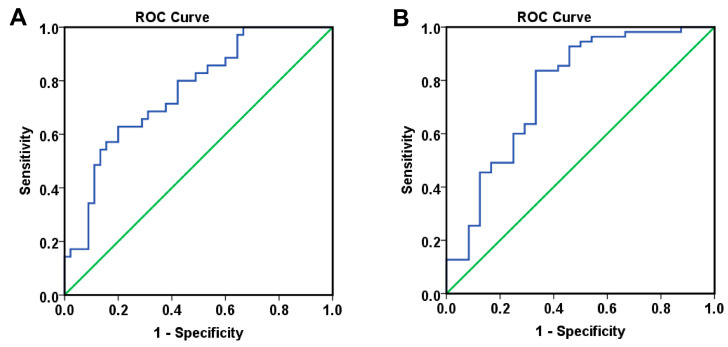
ROC curves resulted from Model_1 for predicting the 24 months death risk measured from (**A**) diagnosis (OS: AUC = 0.760, *p* < 0.001) and (**B**) treatment initiation (OS-TI: AUC = 0.766, *p* < 0.001).

Based on the independent prognostic factors identified through multivariate Cox analysis, we subsequently developed a logistic regression model using survival status as the endpoint (Table 6 and Table S1). Importantly, when computing the 24-month risk of death from either diagnosis or treatment initiation, the AUROC analysis demonstrated good discriminatory ability, with an AUC exceeding 0.760 (*p* < 0.001). This indicates that the evaluated model was able to reliably differentiate between patients at higher versus lower risk of death within 24 months (Table 5, Figure 3).

This model, incorporating the significant factors, also demonstrated good discrimination between overall survivors and non-survivors, with a predicted death probability above 50% effectively separating the groups in the Kaplan–Meier survival curves (*p* < 0.001 for both OS and OS-TI, Figure S2).

We further analyzed the relationship between these biomarkers and CypA in the PDS subgroup, given the previously demonstrated association of this marker with OS. Interestingly, in this subgroup of patients, extremely low values (lower than the 25th percentile) of VEGF-A and VEGF-D were significantly associated with an improvement in OS (Figure 3). Thus, in the multivariate Cox analysis, these two biomarkers were classified according to the 25th percentile values, the other biomarkers being classified according to the median value. The results of this analysis showed that VEGF-A, VEGF-C, VEGF-D and PlGF correlate with OS in addition to CypA. Low levels of VEGF-A, VEGF-D, and CypA, combined with increased levels of VEGF-C and PlGF, were associated with a reduced risk of death (Table 7).

**Figure 4:**
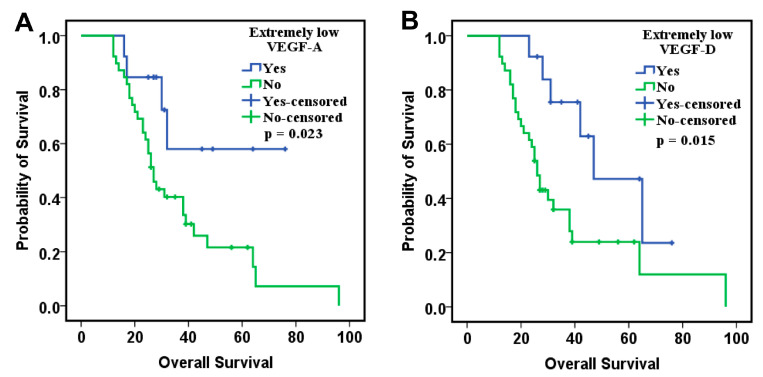
Kaplan–Meier curves of overall survival for patients with (**A**) extremely low VEGF-A values and (**B**) extremely low VEGF-D categorized using their 25th percentile cutoff values.

In both analyses, regardless of the adjustment factors, lower levels of VEGF-A and VEGF-D remained significantly associated with improved OS (Figure 4).

**Figure 5:**
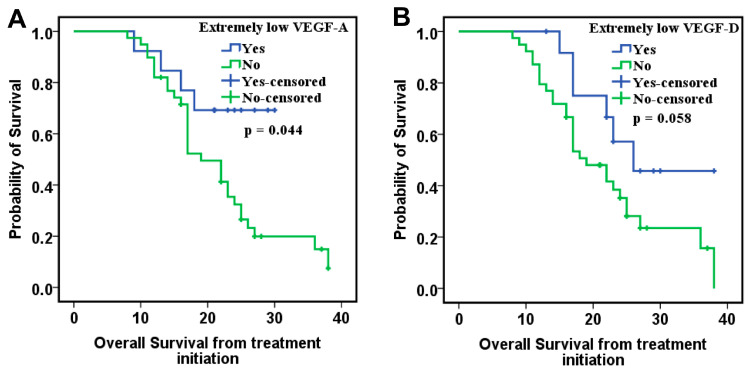
Kaplan–Meier curves of overall survival from treatment initiation for patients with (**A**) extremely low VEGF-A values and (**B**) extremely low VEGF-D categorized using their 25th percentile cutoff values.

In the analysis of overall survival from treatment initiation, patients with initially lower levels of VEGF-A and VEGF-D also exhibited a trend toward improved survival by Cox regression analysis adjusted for time-dependent covariates, including proteinuria and hypertension episodes (Figure 5).

**Figure 6:**
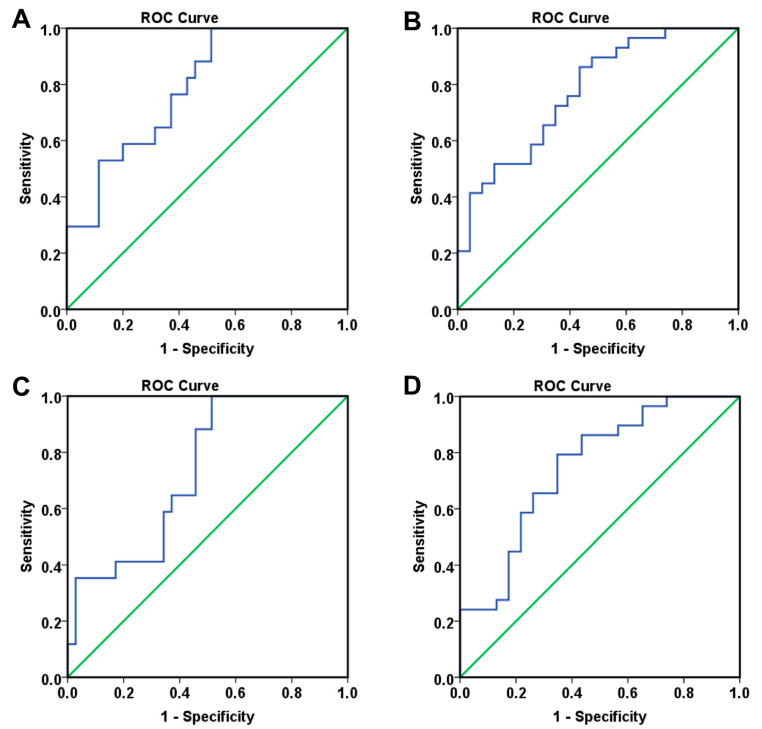
ROC curves for predicting the 24 months death risk measured for Model_2 from (**A**) diagnosis (OS: AUC = 0.787, *p* = 0.001) and (**B**) treatment initiation (OS-TI: AUC = 0.775, *p* = 0.001), and for Model_2.1 from (**C**) diagnosis (OS: AUC = 0.733, *p* = 0.007) and (**D**) treatment initiation (OS-TI: AUC = 0.750, *p* = 0.002).

Next, by including the significant independent predictors in a logistic regression model (Table 8, Tables S2 and S3), we were able to define two distinct approaches: one based on categorizing extremely low levels of VEGF-A and VEGF-D, and another using the continuous dataset. Both approaches demonstrated high prognostic capability for 24-month mortality from diagnosis or treatment initiation, yielding an AUC greater than 0.733 (Table 7, Figure 6).

We also noticed that individuals with a predicted probability of death below 50% had significantly better overall survival, as demonstrated by the Kaplan–Meier curves (*p* = 0.001 for OS, *p* = 0.003 for OS-TI, Figure S3).

#### 2.4.3. The Predictive Role of Biomarkers

To assess the predictive role of biomarkers, we performed a multivariate Cox analysis, this time using PFS as the dependent variable, unlike the previous assessment. In the ITT group, no statistically significant association was identified between the proposed biomarkers and PFS.

**Figure 7:**
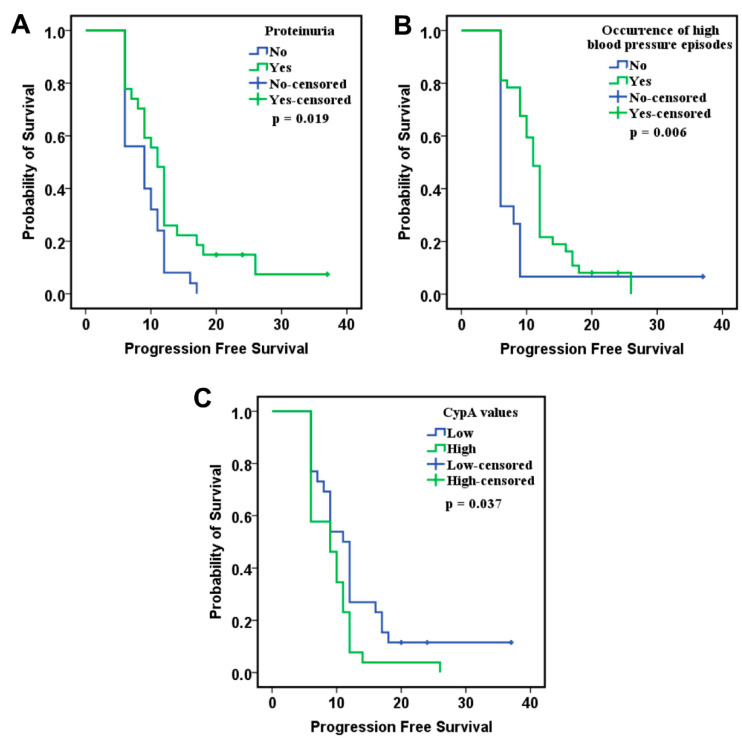
Kaplan–Meier curves of PFS in relation to (**A**) proteinuria, (**B**) occurrence of high blood pressure episodes during therapy and (**C**) CypA levels measured after six months of treatment.

In the PDS subgroup, Tie2 demonstrated a significant correlation with PFS, in the applied model. In addition to the biomarkers, factors associated with PFS from Kaplan–Meier analysis were included, such as the development of proteinuria (Figure 7A), the occurrence of high blood pressure episodes during therapy (Figure 7B), and CypA levels measured after six months of treatment (Figure 7C). Patients with low Tie2 values had a 58.3% lower risk of disease progression compared to patients with elevated values (Table 9). In addition, higher levels of VEGF-C showed a tendency to be associated with a reduced risk of disease progression, but without reaching statistical significance. Other significant factors influencing disease progression included the development of high blood pressure episodes during therapy, associated with a 69.2% reduction in the risk of disease progression, and low CypA values after six months of therapy, which were correlated with a 57.3% reduction in risk.

**Figure 8:**
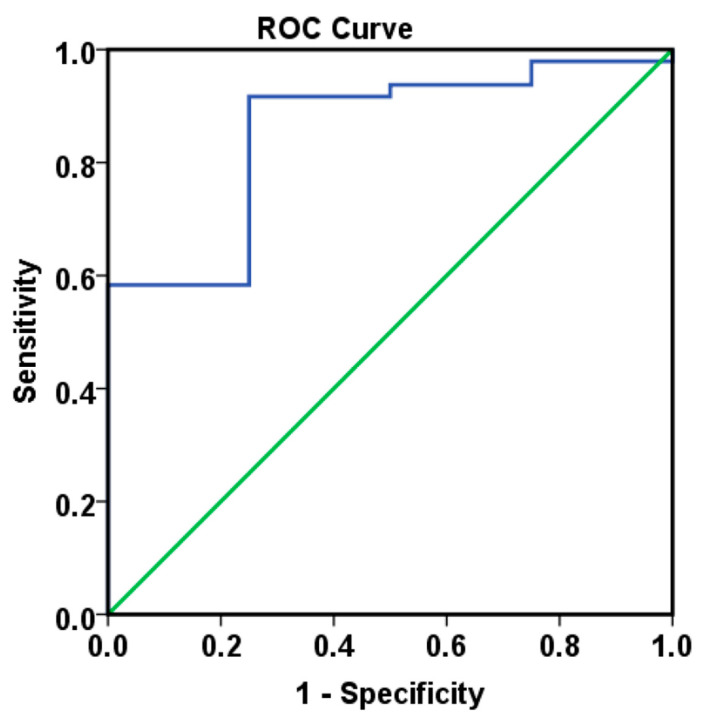
ROC curves resulting from Model_3 for predicting the 18 months progression risk measured (AUC = 0.854, *p* = 0.02).

Furthermore, by including baseline Tie2 levels, CypA levels at 6 months, and the occurrence of high blood pressure episodes after treatment initiation in our analysis, we successfully developed a logistic regression model to estimate the individual probability of disease progression (Table 10 and Table S4). The model demonstrated strong predictive performance for 18-month progression risk, achieving an AUC of 0.854 (*p* = 0.020, Figure 8).

A cutoff value of 90% for the predicted probability effectively discriminated between patients with and without disease progression, as shown in the Kaplan–Meier curves (*p* = 0.002, Figure S4).

The Pearson chi-square test demonstrated a significant difference in radiological tumor response distribution among subgroups defined by median VEGF-C values, supporting the hypothesis of an association between VEGF-C expression and treatment response. Higher levels of VEGF-C were associated with a positive imagistic treatment response and correlated with better treatment outcomes (Table 11).

The integration of multiple angiogenesis-related biomarkers with critical clinical variables (including regional lymph node involvement, metastatic burden, primary tumor resection status, and presence of proteinuria or high blood episodes after treatment initiation) facilitated the development of personalized prognostic models for survival prediction in patients with colorectal cancer treated with bevacizumab.

## 3. Discussion

In colorectal cancer, angiogenesis facilitates tumor progression by sustaining growth and enabling dissemination to distant sites. Targeting this process with antiangiogenic therapies has significantly improved patient outcomes. However, lack of imagistic response to treatment remains a major challenge, limiting long-term efficacy. Thus, elucidating the mechanisms underlying angiogenesis and therapeutic resistance is key to refining treatment strategies. Furthermore, identifying reliable predictive and prognostic biomarkers could facilitate optimal patient selection for antiangiogenic therapy initiation, allowing for a more personalized and effective approach.

Our study investigates the involvement of eight angiogenic factors in the angiogenesis process in CRC and explores the role of circulating biomarkers in optimizing therapeutic decision-making. The study cohort included 80 patients, but CypA values were available for only 52 of them. The results showed that VEGF-A, VEGF-D, and bFGF levels correlated with OS in the ITT group. Patients with low levels of VEGF-A and VEGF-D and high levels of bFGF had the best survival, suggesting that these markers may serve as favorable prognostic factors. Moreover, when CypA was added to the analysis model in the PDS group, along with clinical prognostic factors, low levels of VEGF-A, VEGF-D, and CypA, combined with high levels of VEGF-C and PlGF, were significantly associated with improved OS.

Our results regarding the prognostic role of circulating VEGF-A are consistent with other studies in the literature [8,9,22], as summarized in Table 12. Additionally, existing data on tumor-tissue angiogenesis biomarker expression have shown concordant results with circulating biomarkers, further reinforcing their prognostic value, as also demonstrated in our study (Table 13).

A retrospective analysis of four clinical trials evaluating the prognostic and predictive values of circulating VEGF-A levels in 1816 patients with CRC, lung cancer, and renal cell carcinoma treated with bevacizumab and chemotherapy showed that circulating VEGF-A has a prognostic role, but it was not predictive for the benefit of bevacizumab-based treatment [22]. Results from another more recent study, including 715 patients with metastatic CRC who received chemotherapy with either bevacizumab or cetuximab, showed that VEGF-A and PlGF were prognostic markers for OS, but not for PFS. Additionally, higher levels of VEGF-A were indicative for a higher risk of death, regardless of the associated treatment [8].

In fact, studies in the literature have shown that VEGF-A is an extremely important factor in vascular development, and angiogenesis is indispensable for tumor transformation and progression [22,33,34]. As a result of high levels of VEGF-A expression, malignant cells can maintain their regenerative capacity, leading to a dedifferentiated phenotype and promoting metastasis. The pathophysiological mechanisms [22,33,34,35] highlight VEGF-A as a key target for antiangiogenic therapy, such as bevacizumab, a monoclonal antibody that inhibits VEGF-A activity and tumor angiogenesis.

Although bevacizumab has provided substantial benefits in metastatic CRC treatment [36,37], tumor response to antiangiogenic therapy remains variable among patients. Resistance to antiangiogenic therapy is a common challenge in cancer treatment and most commonly occurs due to alternative angiogenic escape mechanisms that activate signaling pathways independent of VEGF. A potential resistance mechanism involves the overexpression of VEGF-D, which can activate compensatory signaling within the VEGF pathway through VEGFR2, counteracting the effects of VEGF-A inhibition by bevacizumab. Consequently, patients with elevated VEGF-D levels may derive limited therapeutic benefit from bevacizumab due to the presence of an alternative proangiogenic signaling pathway [8].

Interestingly, VEGF-A is overexpressed in CRC, particularly in tumors that have developed distant metastases. Its mRNA expression is increased in both tumor tissue and plasma of CRC patients compared to healthy individuals [9]. An analysis of the expression levels of VEGF-A and its two soluble receptors in two independent CRC cohorts revealed that high VEGF-A expression correlated with a shorter time to disease progression, suggestive for its role as a negative prognostic factor. Moreover, combined overexpression of VEGF-A, VEGFR1, and VEGFR2 conferred a very poor prognosis [9]. In our study, CRC patients with VEGF-A levels above the 25th percentile had significantly lower OS in the PDS cohort. In addition, patients with elevated VEGF-A levels exhibited a higher risk of death in both the ITT and PDS cohorts.

Overall, VEGF-A reflects the angiogenic activity of colorectal cancer, and high levels are consistently associated with adverse outcomes. As a circulating factor, it has prognostic value and may aid in patient risk stratification. When considered together with other angiogenic markers, it could further strengthen clinical decision-making.

VEGF-D is another factor identified in our study with prognostic significance. Patients with low VEGF-D levels had significantly better OS in the PDS cohort and presented a lower risk of death in both the ITT and PDS cohorts, along with other markers and clinicopathological factors included in the analyzed model.

There are studies in the literature that have shown that increased VEGF-D expression is correlated with a poor prognosis and a more aggressive tumor evolution [23,27,28,29,38]. In ovarian cancer, the expression of VEGF-A, VEGF-D and VEGFR1 proteins was higher in metastases compared to primary tumors [27]. Another study analyzed the changes in the expression profile of VEGF-C, VEGF-D and VEGFR3, involved in lymphangiogenesis in endometrial cancer and the results showed that VEGF-D and VEGFR-3 were highly elevated compared to the control group. This change may indicate that, as the endometrium undergoes dedifferentiation, VEGF-D-dependent processes are intensified in malignant cells [28]. In gastric cancer, VEGF-A and VEGF-D were significantly overexpressed in tumor tissue compared to the stroma. Patients with high VEGF-A levels had significantly worse overall survival (OS) than those with low levels. A similar trend was seen for VEGF-D, with higher concentrations associated with a tendency toward poorer OS [38].

In CRC, Ose et al. found that doubling VEGF-D levels was linked to a threefold increased risk of death from rectal cancer, with no significant association in colon cancer [23]. Additionally, VEGF-D has been demonstrated to correlate with PFS in metastatic CRC patients treated with chemotherapy and bevacizumab as first-line therapy [29]. Patients with low VEGF-D expression in the tumor tissue showed the greatest benefit from treatment, both for OS and PFS. In another more recent study, Nixon et al. analyzed a panel of 24 soluble proteins with potential prognostic role in the plasma of patients with metastatic CRC who received chemotherapy in combination with either bevacizumab or cetuximab. The results of this study showed that high levels of VEGF-D were predictive of a lack of benefit on PFS following bevacizumab therapy, in addition to PlGF [8]. Furthermore, patients with extremely low levels of VEGF-D, values below the 25th quartile, had better OS and PFS. These results correlate with the results of our study. Patients with extremely low levels of VEGF-D had the most favorable OS.

An important aspect is the relationship between VEGF-D and disease progression in CRC patients treated with bevacizumab. Lieu et al. demonstrated that circulating VEGF-D and PlGF levels increased at the time of imaging-confirmed progression [24]. This increase suggests a compensatory mechanism through which tumors reactivate angiogenesis to counteract the effects of VEGF-A inhibition, thereby avoiding therapeutic control and promoting malignant progression. The data highlight the role of VEGF-D as a potential prognostic biomarker and its involvement in the mechanisms of resistance to antiangiogenic treatment [8,24].

On the other hand, Taniguchi et al. obtained surprising results regarding the predictive role of VEGF-D for second-line antiangiogenic therapy in metastatic CRC [25]. Patients with high VEGF-D levels had a longer PFS and OS following ramucirumab and chemotherapy; however, statistical significance was achieved exclusively for PFS. The results are contradictory to those obtained in first-line bevacizumab treatment, in which patients with low VEGF-D levels had a better prognosis. Unlike bevacizumab, which only inhibits VEGF-A, ramucirumab (a monoclonal antibody that targets VEGFR2) prevents signaling at the receptor level, regardless of the activating ligand, such as VEGF-A, VEGF-C or VEGF-D [39]. This strategy is important in the context of resistance to bevacizumab therapy, where tumors can compensate by overexpressing VEGF-D or other ligands that continue to activate VEGFR2 [33,40].

VEGF-D exhibits a consistent pattern in colorectal cancer, where very low circulating or tissue levels are associated with favorable prognosis. This suggests that VEGF-D could serve as a negative prognostic biomarker and may help identify patients more likely to benefit from bevacizumab therapy.

bFGF belongs to the FGF family and is involved in cell proliferation, angiogenesis, and metastasis through interaction with its four tyrosine kinase receptors, acting as a proangiogenic factor. In CRC, data from studies show that elevated levels of bFGF are associated with more aggressive tumors and an increased risk of recurrence [12,13,30]. One study analyzed bFGF expression levels in patients with various forms of cancer, including colon cancer, to assess their association with clinicopathological characteristics of the neoplasms [30]. The results showed that elevated levels of bFGF protein expression were associated with more aggressive tumors. Another study showed significant differences in bFGF levels between colorectal cancers in stages I-IIIb compared with those in stage IV, suggesting an association between elevated levels of this factor and tumor progression. Significant differences were also observed between cases with minimal and moderate lymphatic invasion, indicating a possible link between elevated bFGF expression and the ability of the tumor to invade the lymphatic system [13]. However, there is contradictory data regarding the negative prognostic role of elevated bFGF levels in CRC [26]. Kasurinen et al. classified CRC patients into four phenotypic subtypes, immune, canonical, metabolic, and mesenchymal, based on the immunohistochemically determined CD3-CD8 index in tumor and stroma, proliferation index, and stroma-tumor ratio. They evaluated serum levels and tissue expression of angiogenic factors VEGF, bFGF, and PDGF in each subtype. Surprisingly, in the metabolic subgroup, higher serum concentrations of all three markers were associated with significantly improved survival. Additionally, high initial serum bFGF levels proved to be favorable prognostic factors in the canonical subtype [26]. This observation contrasts with other studies that associate high levels of bFGF with a poor prognosis, highlighting the complexity and heterogeneity of CRC. Importantly, our findings are in agreement with the results reported in the aforementioned study, supporting the association of elevated bFGF levels with a favorable prognosis. A possible explanation may be related to the fact that a bFGF-stimulated angiogenesis process may favor a more efficient immune tumor microenvironment, which may inhibit tumor growth. There are data suggesting that FGF can modulate the tumor microenvironment being directly associated with infiltration of M2 macrophages and dendritic cells, which would explain a possible association with a favorable prognosis in CRC [12,41,42].

Another surprising finding in our study was that increased VEGF-C levels correlated with better prognosis and higher disease control rates. In other CRC studies, high levels of VEGF-C were associated with an unfavorable prognosis, being linked to an increased rate of lymph node metastases and lower OS [31,32,43]. A possible explanation for our results may be related to the association of increased VEGF-C expression with different molecular subtypes of CRC, such as those with microsatellite instability or with well-differentiated histological grades, but this is more related to the specific biology of the tumor, not to the direct role of VEGF-C.

CypA is a multifunctional protein involved in protein folding by catalyzing proline bond isomerization, cellular signaling, and modulating inflammatory responses [44]. In cancer, CypA stimulates tumor cell proliferation, angiogenesis, and metastasis by activating pathways such as NF-κB and ERK1/2 [45]. In our previous study, we also showed that lower levels of CypA, both before and after one month of bevacizumab plus chemotherapy, independently predicted better OS and were associated with improved prognosis in metastatic CRC patients [21].

Finally, combining these angiogenic biomarkers with clinical parameters, such as lymph node invasion, and time-dependent variables, including proteinuria, hypertension, and CypA levels at 6 months, allowed the development of logistic regression models that demonstrated good to very good predictive capacity for overall survival, measured from either diagnosis or treatment initiation, as well as for progression-free survival (PFS).

One notable limitation of our study is that tumor angiogenesis was assessed indirectly through circulating biomarkers, rather than by direct tissue-based methods such as immunohistochemistry. Circulating angiogenic factors may not fully reflect the complexity and heterogeneity of the tumor vasculature, which is largely determined by local microenvironmental signals, including hypoxia, stromal interactions, and extracellular matrix remodeling. Nevertheless, systemic angiogenic markers may capture not only local angiogenic activity but also systemic processes, such as the mobilization of endothelial progenitor cells and paracrine signaling influencing vascular remodeling at distant sites. While our approach does not provide a direct histological assessment of angiogenesis, it offers a clinically feasible and biologically relevant perspective that complements tissue-based analyses. Furthermore, future studies could benefit from stratifying patients according to histotype and tumor grading, as these factors may influence angiogenic patterns and clinical outcomes. Together, incorporating both circulating and tissue-level markers, along with stratification by histological features, will be essential to achieve a more comprehensive characterization of tumor angiogenesis. Another limitation of our study is the relatively small cohort size, which may restrict the generalizability of our findings, as well as the lack of external validation. Nevertheless, the results provide valuable insights into the potential role of circulating angiogenic biomarkers, in combination with clinical variables, for predicting survival in bevacizumab-treated colorectal cancer. Importantly, these findings establish a foundation for future research with larger, independent cohorts, which will be essential to validate, refine, and potentially translate these prognostic models into clinical decision-making.

Overall, identifying predictive and prognostic biomarkers for antiangiogenic therapy in colorectal cancer is crucial for optimizing treatment and improving patient outcomes. A deeper understanding of the molecular factors influencing therapeutic response can guide personalized interventions and enhance efficacy. Our study offers new insights into the role of circulating biomarkers, such as VEGF-A, VEGF-D, VEGF-C, bFGF, and CypA, highlighting both novel findings and controversial aspects regarding their significance in this context.

Importantly, by combining multiple parameters identified as independent prognostic factors in multivariate analyses, including both clinical characteristics and paraclinical data, we were able to construct robust mathematical models with high predictive performance for both overall survival (OS) and progression-free survival (PFS). These models demonstrated excellent discriminatory power in stratifying patients according to risk, and allowed for the estimation of individual survival probabilities. This integrative approach supports a more personalized management of patients with colorectal cancer treated with antiangiogenic agents such as bevacizumab, potentially guiding clinical decisions and improving the selection of patients most likely to benefit from therapy.

## 4. Materials and Methods

### 4.1. Patients and Study Design

In this prospective study, we included patients with histopathological confirmed CRC, at least one detectable metastasis, and normal hematological, renal, and hepatic function, who initiated chemotherapy and bevacizumab between May 2019 and January 2021, thereby forming the intention-to-treat (ITT) cohort. We excluded patients with an ECOG performance status >2 and those with conditions that required a delay in the initiation of bevacizumab therapy (e.g., acute ischemic disease, heart failure, or uncontrolled hypertension).

In addition to baseline patient characteristics, such as age, primary tumor location, RAS mutational status, metastatic sites, and the associated chemotherapy regimen, we also evaluated the baseline serum levels of VEGF-A, VEGF-C, VEGF-D, PlGF, FLT-1, bFGF, Tie2 and CypA markers. From the ITT group, we selected a subgroup in which CypA serum levels and their correlations with OS and PFS were previously analyzed in our recent study at baseline and after 6 months of treatment [21], referred to as the PDS group (Partial Data Subset–CypA). Of the 56 patients included in that study, whose data and results have already been published, 4 patients were excluded from the current study cohort due to the impossibility of testing the biomarkers VEGF-A, VEGF-C, VEGF-D, PlGF, FLT-1, bFGF, and Tie2.

We also monitored the occurrence of adverse events, which were classified according to Common Terminology Criteria for Adverse Events v5.0.

The treatment regimen administered to the patients included bevacizumab at a dose of 7.5 mg/kg every 3 weeks or 5 mg/kg every 2 weeks, in combination with chemotherapy. The chemotherapy regimens were based on oxaliplatin (CapeOX, mFOLFOX6), irinotecan (XELIRI, FOLFIRI), or fluoropyrimidine (capecitabine monotherapy or the de Gramont regimen), with doses recommended by international guidelines.

The imaging response was assessed at a minimum of 6 months following treatment initiation, using either CT or MRI, in accordance with the Response Evaluation Criteria in Solid Tumors (RECIST) version 1.1 [46], as interpreted by the attending physician. The objective response rate (ORR) was defined as the proportion of patients achieving either a complete or partial response, while the disease control rate (DCR) encompassed patients with complete response, partial response, or stable disease.

The primary objective of the study was to evaluate OS, defined as the time from diagnosis to patient death, regardless of the cause, in relation to biomarker values and other clinicopathological characteristics within the ITT and PDS groups. Secondary endpoints included PFS, defined as the time from initiation of bevacizumab therapy to either disease progression or death, as well as objective tumor response rate, DCR, and the incidence of adverse events.

Ethical approval was granted by the Ethics Committee of the Regional Institute of Oncology in Iasi, and all participants provided informed consent prior to enrollment.

### 4.2. Analysis of Serum Biomarkers

Fasting blood samples were collected just before the administration of first dose of bevacizumab and chemotherapy. The samples were centrifuged at 2000× *g* for 5 min within the first 6 h of collection, and the serum was stored at −80 °C until further biomarker analysis.

We measured the serum levels of VEGF-A, VEGF-C, VEGF-D, PlGF, FLT-1, bFGF and Tie2 using the chemiluminescence technique according to the manufacturers’ instructions, while for CypA we used ELISA technique, the methodology described in our recent work [21].

### 4.3. Statistical Analysis

Statistical analyses were performed using SPSS v.20.0 (IBM SPSS, Armonk, NY, USA). The basic characteristics of the ITT and PDS patient groups are expressed by frequency, median values and ranges (min–max). Biomarkers were dichotomized, when necessary, with patient populations divided into high (≥median), low (<median) or extremely low (below the 25th percentile) groups for each analyte. OS and PFS were analyzed using Kaplan–Meier curves, and comparisons between patient groups were made with the Log-rank test. Multivariate Cox analyzes were performed to assess the prognostic and predictive role of biomarkers. In order to select the adjustment factors for prognostic models, we assessed the clinicopathological factors associated with OS and subsequently with PFS through Kaplan–Meier analysis, for both the ITT group and the PDS group. To distinguish prognostic from predictive effects in our analyses, OS was used as the time-to-event variable in Cox models to evaluate prognostic value, reflecting the natural course of the disease. PFS was used to assess predictive value, indicating the potential impact of bevacizumab-based therapy on disease progression. This approach allows a clear separation between factors that influence general disease outcome and those that may predict treatment benefit. For the logistic regression models and Cox proportional hazards analyses of overall survival (OS) measured from treatment initiation (OS-TI), as well as progression-free survival (PFS), internal validation was performed using bootstrapping. Model performance, including discrimination and calibration, was assessed using 1000 bootstrap resamples with bias-corrected estimates to account for potential overfitting. The Pearson chi-square test was used to evaluate the associations between subgroups based on various clinicopathological factors.

## 5. Conclusions

The results of our study highlight a profile of angiogenic markers that influence OS in CRC in patients who received antiangiogenic therapy and chemotherapy. Low levels of VEGF-A and VEGF-D, associated with high levels of bFGF, were correlated with improved survival, suggesting that these markers could function as favorable prognostic factors. Furthermore, the integration of CypA into the analysis model, alongside clinically relevant prognostic factors, revealed additional significance: patients with low levels of VEGF-A, VEGF-D, and CypA, combined with high levels of VEGF-C and PIGF, exhibited significantly improved OS. This combination of biomarkers reflects not only the biological heterogeneity of the tumor, but also the potential of a multifactorial model for patient stratification.

Importantly, our findings highlight the value of integrating both clinical and paraclinical data, including serum biomarkers and treatment-related events, into mathematical models to enhance the personalized prediction of OS and PFS for CRC patients undergoing bevacizumab therapy. By combining dynamic biological markers with patient-specific clinical characteristics, such models allow for more accurate risk stratification and individualized prognostication. This approach not only supports more informed therapeutic decisions but also enables the identification of patient subgroups most likely to benefit from targeted therapies, such as bevacizumab, while minimizing unnecessary exposure to treatment-related toxicity in non-responders. Ultimately, such prognostic and predictive modeling represents a step toward truly personalized oncology care, optimizing outcomes and resource utilization.

## Figures and Tables

**Table 1:** Baseline characteristics of the studied groups.

Characteristic	ITT		PDS	
	Frequency	Percent	Frequency	Percent
Median age (range)	61 (37–82) years		61 (37–82) years	
Gender				
Male	44	55	29	55.8
Female	36	45	23	44.2
Sidedness				
Right	23	28.7	13	25
Left	57	71.3	39	75
Primary tumor resection	56	70	37	71.2
Disease stage at diagnosis				
Metastatic	62	77.5	42	80.8
Non-metastatic	18	22.5	10	19.2
Sites of metastasis				
Liver	34	42.5	24	46.2
Peritoneal	7	8.8	4	7.7
Lung	5	6.2	4	7.7
Multiple	34	42.5	20	38.4
RAS/BRAF status				
Wild type	34	42.5	20	38.4
KRAS mutation	34	42.5	26	50
NRAS mutation	8	10	6	11.6
BRAF mutation	4	5	0	0
Associated chemotherapy				
Oxaliplatin	46	57.5	34	65.4
Irinotecan	25	31.3	15	28.8
Fluorouracil/Capecitabine	9	11.2	3	5.8
Radiological tumor response ^‡^				
Complete response	2	2.5	2	3.8
Partial response	14	17.5	11	21.2
Stable disease	23	28.7	22	42.3
Progressive disease	41	51.2	17	32.7

ITT (Intention-to-Treat population); PDS (Partial Data Subset—CypA); ^‡^ assessed according to RECIST 1.1 criteria, performed after 6 months of treatment.

**Table 2:** Treatment-induced adverse effects.

Event	Any Grade (n, %)	Grades ≥ 3 (n, %)
Anemia	33, 41.3	2, 2.5
Neutropenia	29, 36.3	7, 8.8
Thrombocytopenia	24, 30	1, 1.3
Liver toxicity	35, 43.8	1, 1.3
Proteinuria	32, 40	- *
Digestive toxicity	20, 25	2, 2.5
Neurological toxicity	26, 32.5	1, 1.3

* tested only qualitative.

**Table 3:** Serum biomarker concentrations and their ranges.

Biomarker	25th Percentile	Median Value	75th Percentile	Range (Min–Max)
VEGF-A (pg/mL)	2670	6731	14,810	5.7–47,967
VEGF-C (pg/mL)	80,127	110,178	161,660	17,007–300,523
VEGF-D (pg/mL)	33,916	42,055	58,777	14,474–139,513
PlGF (pg/mL)	188.9	212.2	252.3	121.2–424.5
FLT-1 (pg/mL)	2669	3359	4714	1145–43,279
bFGF (pg/mL)	75.97	212.38	410.7	14.42–1941.53
Tie2 (pg/mL)	57,775	67,363.51	82,814	24,844–252,424
CypA (ng/mL) *	9.35	54.65	112.1	1.54–168.61

* data available only for 52 patients; PDS (Partial Data Subset–CypA).

**Table 4:** The relationship between clinicopathological factors and serum biomarker levels.

	**VEGF-D values**		
	lower than the 75th percentile	higher than the 75th percentile	*p*
**Location of the primary tumor, N (%)**			0.007
Right colon	22 (95.7%)	1 (4.3%)	
Left colon	38 (66.7%)	19 (33.3%)	
	**Tie2 values**		
	lower than the 25th percentile	higher than the 25th percentile	*p*
**Location of the primary tumor, N (%)**			0.015
Right colon	10 (43.5%)	13 (56.5%)	
Left colon	10 (17.5%)	47 (82.5%)	
	**Tie2 values**		
	lower than the 25th percentile	higher than the 25th percentile	*p*
**Tumor invasion of regional lymph nodes, N (%)**			0.030
No	0 (0%)	12 (100%)	
Yes	20 (29.4%)	48 (70.6%)	
	**Tie2 values**		
	lower than the median	higher than the median	*p*
**Resection of the primary tumor, N (%)**			0.051
Yes	24 (42.9%)	32 (57.1%)	
No	16 (66.7%)	8 (33.3%)	

Note: Soluble biomarkers and categorization criteria are presented in bold for emphasis.

**Table 5:** Associations between biomarker levels, clinicopathological factors and OS, using multivariate Cox analysis in the ITT group.

Factor	*p*	HR	95% CI
VEGF-A	0.038	0.543	0.305–0.966
VEGF-C	0.963	1.014	0.555–1.853
VEGF-D	0.022	0.488	0.264–0.901
PlGF	0.909	1.035	0.573–1.870
FLT-1	0.113	0.611	0.332–1.124
bFGF	0.044	1.809	1.015–3.222
Tie2	0.260	1.466	0.754–2.850
Regional lymph node tumor invasion	0.049	0.359	0.130–0.994
Metastatic sites	0.853	1.014	0.873–1.178
Resection of the primary tumor	0.005	0.412	0.221–0.767
Proteinuria	<0.001	0.287	0.151–0.545
**Model**	***p***	**OR**	**95% CI**
Model_1	<0.001	19.805	4.563–85.955
**24 months death risk**	***p***	**AUC**	**95% CI**
Model_1 from diagnosis	<0.001	0.760	0.656–0.864
Model_1 from treatment initiation	<0.001	0.766	0.641–0.891

Note: The mathematical model and the 24 months death risk are presented in bold for emphasis.

**Table 6:** OS logistic regression of biomarkers included in Model_1.

Model_1 (Logistic Regression)	*p*	OR	95% CI
VEGF-A	0.012	1.180	1.037–1.344
VEGF-D	0.309	1.014	0.987–1.042
bFGF	0.675	1.000	0.998–1.002
Regional lymph node tumor invasion	0.021	2.951	1.18–7.382
Resection of the primary tumor	0.399	2.009	0.397–10.173
Proteinuria	0.010	6.277	1.549–25.429

**Table 7:** Associations between biomarker levels and OS, using multivariate Cox analysis in PDS group.

Factor	*p*	HR	95% CI
VEGF-A *	0.006	0.171	0.049–0.606
VEGF-C	0.005	3.532	1.454–8.580
VEGF-D *	0.003	0.192	0.066–0.561
PlGF	0.004	3.919	1.564–9.817
FLT-1	0.961	1.022	0.426–2.455
bFGF	0.265	0.651	0.306–1.385
Tie2	0.386	0.722	0.346–1.508
CypA (baseline)	<0.001	0.163	0.065–0.411
**Model**	***p***	**OR**	**95% CI**
Model_2	0.001	17.929	3.250–98.89
Model_2.1	0.009	6.078	1.568–23.57
**24 months death risk**	***p***	**AUC**	**95% CI**
Model_2 from diagnosis	0.001	0.787	0.663–0.911
Model_2 from treatment initiation	0.001	0.775	0.649–0.901
Model_2.1 from diagnosis	0.007	0.733	0.595–0.870
Model_2.1 from treatment initiation	0.002	0.750	0.614–0.885

* categorized using their 25th percentile cutoff values. Note: The mathematical model and the 24 months death risk are presented in bold for emphasis.

**Table 8:** OS logistic regression of biomarkers included in Model_2.

Model_2 (Logistic Regression)	*p*	OR	95% CI
VEGF-A *	0.004	0.055	0.008–0.396
VEGF-C	0.861	1.001	0.987–1.016
VEGF-D *	0.069	0.207	0.038–1.131
PlGF	0.097	0.985	0.968–1.003
CypA (baseline)	0.035	1.020	1.001–1.038
**Model_2.1 (logistic regression)**	***p***	**OR**	**95% CI**
VEGF-A	0.004	0.055	0.008–0.396
VEGF-C	0.861	1.001	0.987–1.016
VEGF-D	0.069	0.207	0.038–1.131
PlGF	0.097	0.985	0.968–1.003
CypA (baseline)	0.035	1.020	1.001–1.038

* categorized using their 25th percentile cutoff values. Note: The mathematical model is presented in bold for emphasis.

**Table 9:** Associations between biomarker levels, other factors and PFS, using multivariate Cox analysis in PDS group.

Factor	*p*	HR	95% CI
VEGF-A *	0.248	0.624	0.280–1.388
VEGF-C	0.080	0.520	0.250–1.082
VEGF-D *	0.821	1.099	0.486–2.485
PlGF	0.883	1.056	0.511–2.184
FLT-1	0.465	1.303	0.640–2.655
bFGF	0.279	1.481	0.727–3.016
Tie2	0.025	0.417	0.194–0.898
CypA ^a^	0.022	0.427	0.206–0.887
Proteinuria	0.099	0.564	0.285–1.113
High blood pressure episodes	0.005	0.308	0.135–0.706
**Model**	***p***	**OR**	**95% CI**
Model_3	0.016	34.165	1.954–597.4
**18 months progression risk**	***p***	**AUC**	**95% CI**
Model_3	0.020	0.854	0.684–1.000

* categorized using their 25th percentile cutoff values; ^a^ measured after six months of treatment. Note: The mathematical model and the 18 months progression risk are presented in bold for emphasis.

**Table 10:** PFS logistic regression of biomarkers included in Model_3.

Model_3 (Logistic Regression)	*p*	OR	95% CI
VEGFC	0.254	1.017	0.988–1.046
Tie2	0.848	1.059	0.590–1.900
CypA ^a^	0.113	1.023	0.995–1.053
High blood pressure episodes	0.531	0.602	0.123–2.945

^a^ measured after six months of treatment.

**Table 11:** Disease control rate depending on the median level of VEGF-C.

	VEGF-C Levels According to the Median Value		
	Lower N (%)	Higher N (%)	*p*
Disease control rate	15 (38.5)	24 (61.5)	0.044
Progressive disease	25 (61)	16 (39)	

**Table 12:** Overview of circulating angiogenesis biomarkers in cancer.

Author, Journal, Year	Study Type	Cancer Type	Sample Size	Endpoints	Biomarkers	Conclusions	Findings in Our Study
Nixon, A.B.; et al., *Clinical Cancer Research*, 2022 [8]	Prospective	Metastatic CRC	715	PFS, OS	“Angiome” panel–24 soluble plasma protein biomarkers including VEGF-A, VEGF-D, PIGF	↑ PlGF predicts less PFS benefit from bevacizumab; ↑ VEGF-D predicts lack of benefit only in FOLFOX + bevacizumab regimen; PlGF and VEGF-A were prognostic for OS	↓ VEGF-A and VEGF-D were associated with improved OS; ↑ PlGF levels correlated with better OS in the PDS group
Hegde, P.S.; et al., *Clinical Cancer Research*, 2013 [22]	Retrospective exploratory biomarker analysis	Metastatic CRC, lung cancer, and renal cell carcinoma	1816	PFS, OS	Circulating VEGF-A	↑ baseline VEGF-A levels were associated with poor OS	↓ VEGF-A levels were associated with improved OS
Ose, J.; et al., *Cancer Epidemiology, Biomarkers and Prevention*, 2022 [23]	Observational	CRC	426	OS, disease-free survival, recurrence risk	Adhesion molecules, VEGF-A, VEGF-D	↑ VEGF-D levels were associated with a threefold increased risk of death in rectal cancer; VEGF-A levels were not significantly associated with patient outcomes	↓ VEGF-A and VEGF-D levels were associated with improved OS
Lieu, C.H.; et al., *PLoS ONE*, 2013 [24]	Observational	Metastatic CRC	Observational cohort: 42 patients; Validation cohort: 403 patients	Association of VEGF ligands with resistance to bevacizumab and chemotherapy	VEGF-A, VEGF-C, VEGF-D, PlGF	Circulating VEGF-D and PlGF were increased at disease progression; VEGF-A and VEGF-C are not predictive for outcomes or resistance	↓ VEGF-A and VEGF-D levels were associated with improved OS; ↑ PlGF and VEGF-C levels correlated with better OS in the PDS group
Taniguchi, H.; et al., *Current Medical Research and Opinion*, 2021 [25]	Observational	Metastatic CRC	1072	OS, PFS	VEGF-D	↑ VEGF-D is associated with greater OS and PFS benefit for second-line therapy	↓ VEGF-D levels were associated with improved OS
Jibiki, N.; et al., *International Surgery*, 2014 [13]	Observational	CRC	92	Association of bFGF levels with clinicopathological factors	bFGF	↑ bFGF levels are associated with advanced cancer stages and lymphatic invasion	↑ bFGF levels are associated with favorable prognosis
Kasurinen, J.H.; et al., *Cancers*, 2023 [26]	Observational	CRC	322	Disease-specific survival	VEGF, bFGF, PDGF-bb (serum and tissue)	bFGF prognostic value depends on CRC phenotypic subtype	↑ bFGF levels are associated with favorable prognosis

Note: ↑increased; ↓ decreased.

**Table 13:** Overview of tumor tissue angiogenesis biomarkers in cancer.

Author, Journal, Year	Study Type	Cancer Type	Sample Size	Endpoints	Biomarkers	Conclusions
Zhang, S.D.; et al., *OncoTargets and Therapy*, 2015 [9]	Observational	CRC	200	OS, PFS	VEGF-A, VEGFR1, VEGFR2	↑ VEGFA, VEGFR1, and VEGFR2 expression predicts poor prognosis and low bevacizumab response
Sopo, M.; et al., *BMC Cancer*, 2019 [27]	Observational	Ovarian cancer	86 primary tumor samples + 16 matched distant metastases	PFS and OS, primary–metastasis expression analysis	VEGF-A, VEGF-C, VEGF-D, VEGFR1, VEGFR2, VEGFR3	Metastases show ↑ VEGF-A, VEGF-D, and VEGFR1 than primaries; ↓ VEGF-A in primary tumors predicts poor OS; ↑ VEGF-C and ↓ VEGFR3 predict shorter PFS
Oplawski, M.; et al., *Current Pharmaceutical Biotechnolog*, 2019 [28]	Retrospective	Endometrial cancer	60	Correlation with tumor grade, clinicopathological features, and prognosis	VEGF-C, VEGF-D, VEGFR3	↑ VEGF-C, VEGF-D, and VEGFR3 expression correlates with higher grade
Weickhardt, A.J.; et al., *British Journal of Cancer*, 2015 [29]	Retrospective	CRC	268 tumor specimens	OS, PFS, response to Bevacizumab	VEGF-D	↑VEGF-D predicts poor outcomes; ↓ VEGF-D indicates bevacizumab benefit
Liu, M.; et al., *Oncology Letters*, 2017 [30]	Retrospective	Lung, breast, colorectal cancer, melanoma	508	Association with metastasis and clinicopathological features	bFGF	↑ bFGF correlates with metastasis and advanced tumor stages
Mazeda, I.; et al., *Gastrointestinal Disorders*, 2020 [31]	Prospective	CRC	210	OS, lymph node metastasis, recurrence	VEGF-A, VEGF-C, VEGF-D, VEGFR2, VEGFR3	VEGF-A, VEGF-C, and VEGFR2 are significantly higher in metastatic nodes and associate with poor prognosis
Gao, M.; et al., *Oncotarget*, 2016 [32]	Observational	CRC	108	OS, lymph node metastasis, tumor stage	VEGF-C, eIF4E, E-cadherin, MMP-2	↑ eIF4E, VEGF-C, and MMP-2, and ↓ E-cadherin, are associated with advanced tumor features and poor prognosis

Note: ↑increased; ↓ decreased.

## Data Availability

All data generated or analyzed in this study are included in this article and the accompanying Supplementary Information files.

## Supplementary Materials

The following supporting information can be downloaded at: https://www.mdpi.com/article/10.3390/ijms26199332/s1.

## Abbreviations

The following abbreviations are used in this manuscript:

OS	Overall survival
OS-TI	Overall survival from treatment initiation
PFS	Progression-free survival
VEGF-A	Vascular endothelial growth factor A
VEGF-C	Vascular endothelial growth factor C
VEGF-D	Vascular endothelial growth factor D
PlGF	Placental Growth Factor
FLT-1	VEGFR1 (VEGF receptor 1)
bFGF	Basic Fibroblast Growth Factor
Tie2	Tyrosine Kinase with Immunoglobulin-like and Epidermal Growth Factor-like Domains 2
CypA	Cyclophilin A
ITT	Intention-to-Treat population
PDS	Partial Data Subset
CRC	Colorectal cancer
