# Supplementary material for: Predicting Survival in Bevacizumab-Treated Colorectal Cancer: Personalized Mathematical Models Based on Clinical and Angiogenic Biomarkers
Source: Int J Mol Sci. 2025 Sep 24;26(19):9332. doi: 10.3390/ijms26199332 (PMC12525502; doi:10.3390/ijms26199332)
Supplement: Supplementary file 1 [file ijms-26-09332-s001.zip › Supplementary Figures S1-S4.pdf]

Supplementary Figures S1-S4

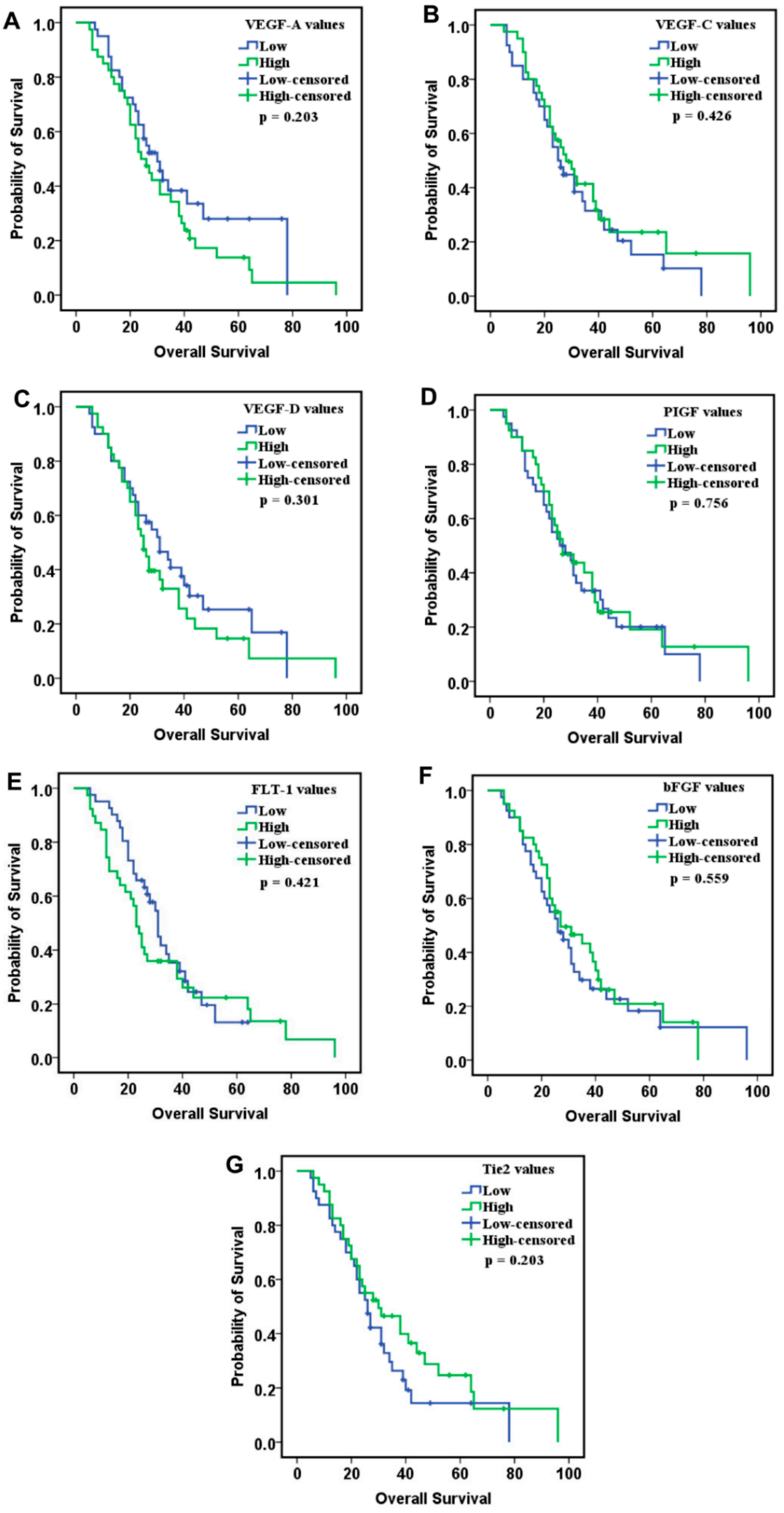

Figure S1. Kaplan–Meier curves of overall survival depending on median values of **(A)** VEGF-A, **(B)** VEGF-C, **(C)** VEGF-D, **(D)** PIGF, **(E)** FLT-1, **(F)** bFGF and **(G**) Tie2.

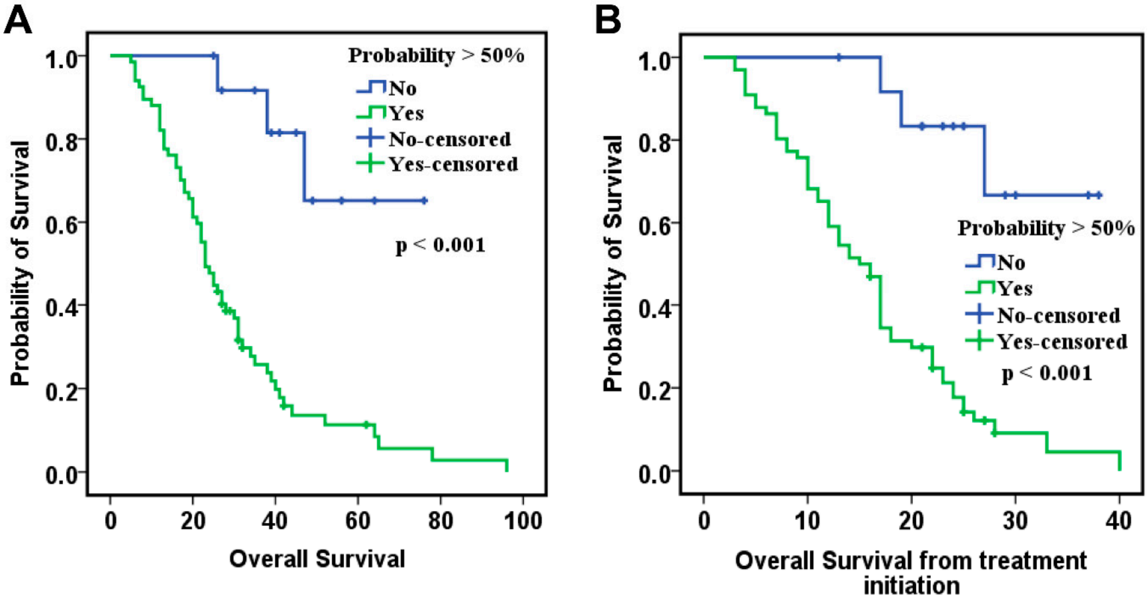

Figure S2. Kaplan–Meier curves of overall survival for patients stratified based on the death prediction probability given by Model_1 (≤50% vs. >50%) measured from **(A)** diagnosis and **(B)** treatment initiation.

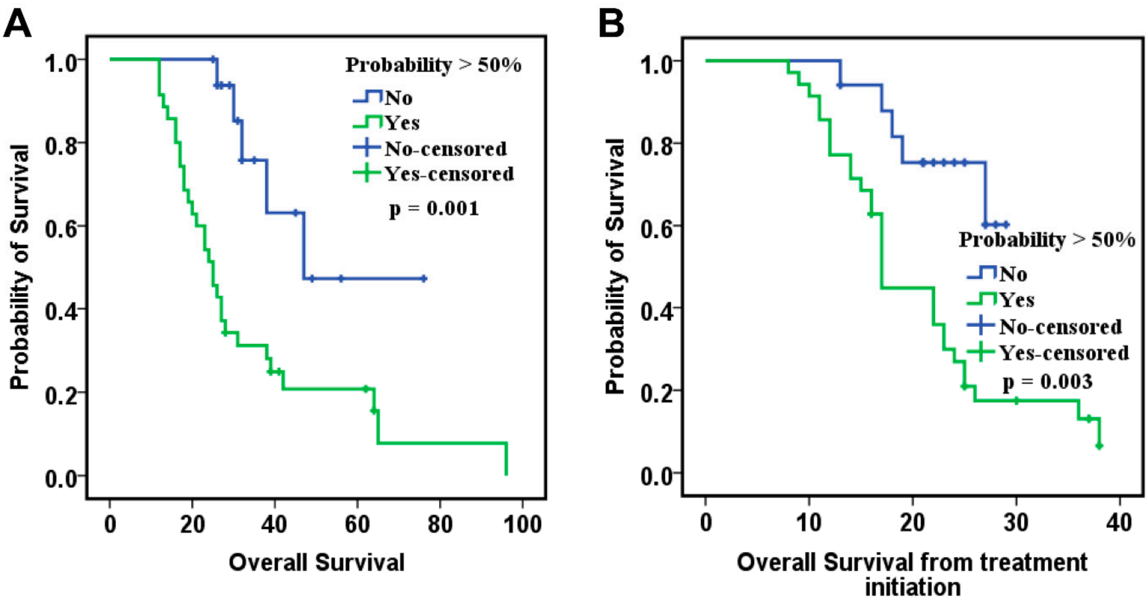

Figure S3. Kaplan–Meier curves of overall survival for patients stratified based on the death prediction probability given by Model_2.1 (≤50% vs. >50%) measured from **(A)** diagnosis and **(B)** treatment initiation.

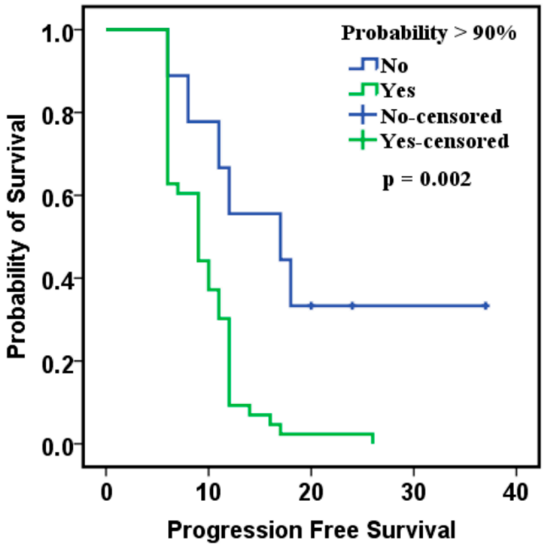

Figure S4. Kaplan–Meier curves of progression free survival for patients stratified based on the disease progression probability given by Model_3 (≤90% vs. >90%).
